# Nutrition of Six Selected Neo-Tropical Mammals in Trinidad and Tobago with the Potential for Domestication

**DOI:** 10.3390/vetsci5020052

**Published:** 2018-05-14

**Authors:** Kavita Ranjeeta Lall, Kegan Romelle Jones, Gary Wayne Garcia

**Affiliations:** The Open Tropical Forage-Animal Production Laboratory (OTF-APL), Department of Food Production (DFP), Faculty of Food and Agriculture (FFA), The University of the West Indies (UWI), St Augustine, Trinidad and Tobago; kavita.lall1@my.uwi.edu (K.R.L.); prof.gary.garcia@gmail.com (G.W.G.)

**Keywords:** agouti (*Dasyprocta leporina*/*D. aguti*), lappe/paca (Cuniculuspaca/Agouti paca), capybara (*Hydrochoerus hydrochaeris*), manicou/opossum (*Didelphis marsupial insularis*), collared peccary (*Tayassu tajacu*), red brocket deer *(Mazama americana*)

## Abstract

This review highlights the available literature on the nutrition of six neo-tropical animals with the potential for domestication—the agouti (*Dasyprocta leporina*/*D. aguti*), lappe (*Agouti paca*), capybara (*Hydrochoerus hydrochaeris*), manicou/opossum (*Didelphis marsupialis insularis*), collared peccary (*Peccary tajucu*) and the red brokcet deer (*Mazama americana*). Over 100 references were used, spanning over 100 years. The earliest being 1915 and the most recent being 2018. The references used in this review were synthesized to give a detailed look of the dentition, anatomy of the gastrointestinal tract and type of feed these animals consume. Nutritional requirements of the animals are required to understand what is needed for growth, maintenance and reproduction of each physiological stage. The agouti (*D. leporina/D. aguti*) was observed to be a monogastric mammal that fed primarily on fruits, seeds, animal matter and practiced caecotrophy. The lappe/paca (*C. paca/A. paca*) was described as a strict herbivore and a frugivore which practiced caecotrophy, with a diet that varied throughout the year, according to food availability. The capybara (*H. hydrochaeris*) was found to be the largest known rodent and was described as a semiaquatic hindgut fermenter that practiced caecotrophy. The manicou/opossum (*D. marsupialis insularis*) was found to be an omnivore with a simple stomach. The collared peccary (*T. tajacu*) was found to be frugivorous. Their unique stomach enabled them to consume a wide variety of feedstuff, allowing them to be found in a wide range of habitats. The red brocket deer (*M. americana*), a ruminant, was described as a browser that consumed mainly fruits and seeds and they frequented mineral lick. Knowledge of what they consume in the wild is important, so that we know what to feed in captivity. There is also the need to evaluate captive diets while trying to domesticate these mammals and develop nutrient requirement tables for these neo-tropical animals. Finally, an understanding of the dentition and gastrointestinal tract is important to increase efficiency (nutritional and cost). These six neo-tropical mammals were chosen due to their prevalence as game species in Trinidad and Tobago.

## 1. Introduction

According to World Health Organization, nutrition is the consumption of food to meet the body’s dietary requirements. A well-balanced diet constitutes good nutrition, while an imbalanced diet or poor nutrition contributes to reduced immunity, decreased physical and mental development and reduced productivity [1]. More specifically, in the animal’s basal diet, amino acids (essential and nonessential), carbohydrates, fatty acids, minerals, water and vitamins should be provided in the feed. Good quality water is also important because it affects feed intake. Proper nutrition is needed for optimal performance at all physiological stages of animal and it is important to know requirement for specific physiological stages [2].

Nutritional ecology evaluates the link between the environment and an organism’s nutritional requirement, foraging behavior and utilization of nutrients [3]. The concept of nutritional ecology encompassed nutrition, organism and ecology [4]. A new concept, called the Geometric Framework, integrates nutrition with the biological requirement of organism. It was stated that animals require many different nutrients in changing amounts and balance for growth and maintenance [5].

Animals can be classified as generalists (omnivores) or specialists (herbivores and carnivores). Generalists eat a wide variety of feedstuff, while specialists consume one or very few types of feedstuff. Omnivores have a wide diet consisting of plant and animal material. Herbivorous animals only consume plants, with different herbivorous animals consuming specific parts of the plant, for example leaves, fruits or nectar or a combination of two. Those that consume leaves require strong teeth and large stomachs. Carnivorous animals refer to those that consume only meat [6]. These animals have evolved dentition and digestive systems to suit their type of diets. Herbivores that consume plants have strong, flat molars to grind leaves efficiently, while having small to non-existent canines as they do not tear meat. Some herbivores even have chisel-like incisors to gnaw through wood and seeds, while some do not have incisors on the upper jaw. The diastema is necessary to provide space for repositioning of plant material as it is chewed. Herbivores could be further classified as grazers (feeding on grasses near the ground) or browsers (consumers of leaves, shoots and twigs) or both. Since carnivores eat meat, or animal material, they do not require such broad, flat molars. Instead, they usually hunt and kill their food, so they are well equipped with sharp incisors and pointed canine teeth and fewer molars, which have serrated edges. Omnivores consume both plant and animal matter and can have either heterodont or homodont dentition. Heterodonts have incisors and canines for tearing and molars for grinding, while homodonts have teeth of more or less the same size and shape, as they are used to obtain food [6].

Digestibility of feed is a critical factor when investigating the type of feed for animals. Digestibility is defined as the amount of nutrient that can be absorbed from the feedstuff consumed [7]. The type of diet is also reflected in the digestive system of the animals. Since meat is easier to digest than fiber (found in plant material), carnivores have a shorter and simpler digestive system as compared to herbivores. Carnivores are strictly monogastric animals (one stomach), with small caeca, as compared to herbivores which have either a monogastric stomach with an enlarged, fully functional caecum or a four-chambered stomach with a large rumen (ruminants). They can also be classified as foregut fermenters (ruminant herbivore) or hindgut fermenters (non-ruminant herbivore). Herbivores have special microflora, found in the caecum or rumen, which breaks down cellulose into volatile fatty acids and glucose. Since omnivores eat both plant and animal matter, their digestive system is similar to carnivores except that the caecum is functional, just not as large and efficient as in herbivores [8].

Wildlife farming is the concept where these animals are reared for human consumption. It serves two functions: (1) providing meat for human consumption; and (2) rearing animals in captivity to be reintroduced into the wild. If these animals are to be reared in captivity, factors affecting their production become important. Of these factors, feeding and nutrition must be mastered to keep the animals alive and allow them to reproduce in captivity. The agouti (*D. leporina/D. aguti*) was found to be a monogastric mammal that feeds primarily on fruits and seeds, with some consumption of animal matter. They were described as having scatter-hoarding behavior, which contributed to them being short distance seed dispersers and they also exhibited coprophagy [9].

The lappe/paca (*C. paca/A. paca*) was described as a strict herbivore and a frugivore, with a diet that varied throughout the year, according to food availability. It was found to be a monogastric mammal that exhibited caecotrophy [10]. The capybara (*H. hydrochaeris*) was found to be the largest known rodent and was described as a semi-aquatic herbivore. They were found to be hindgut fermenters that practiced caecotrophy [11]. The manicou/opossum *(D. marsupialis insularis*) was found to be an omnivore with a simple stomach. The collared peccary (*T. tajacu*) was found to be frugivorous. Their unique stomach enabled them to consume a wide variety of feedstuff, allowing them to be found in a wide range of habitats. The red brocket deer (*M. americana*), a ruminant, was described as a browser that consumed mainly fruits and seeds and they frequented mineral licks. The purpose of this literature review was to compile information regarding, not only the feedstuff of the six different neo-tropical animals listed, but also their dentition and digestive system. Knowledge of the diet and feeding of these neo-tropical mammals will improve the quality of conservation, rehabilitation and veterinary care. Veterinarians will be able to advise clients as well as treat neo-tropical animals that suffer from nutritional deficiency diseases in captivity. 

## 2. Agouti (*D. leporina/D. aguti*)

### 2.1. Eating Habits and Feed Type

The agouti is classified as a herbivore, which feeds during the early morning and evening in the wild, but in captivity could adapt to different feeding times. In the wild, they consumed roots of ferns, tubers, succulent plants, bark, fruits, berries, seeds and nuts [12]. In captivity, they could eat a wide variety including coconut, bread and cooked rice. The stomach contents were quantified and their diet consisted of 82.2% fruit (pulp, 37.6%; and seeds, 44.6%), 9.3% animal matter, 6.2% fiber and 2.3% leaves. They mostly ate pulp (64.8%) when fruits were abundant, but, when fruiting was low, they turned to seeds and cotyledons (73.0%) and animal matter (16.4%) With the high percentage of fruit found in the stomach content, the agouti was classified as a frugivore [13]. Similar findings were seen where the diets were supplemented with insects, seeds, leaves and fiber when less fruits were available [14] (Table 1). During the period of fruit bearing, they ate the pulp rather than the seeds [15]. The agouti was documented as the only known mammal that could gnaw through the Brazil nut’s (*Bertholletia excelsa*) tough pericarp [16,17,18,19].

In captivity, dusty materials should be avoided when feeding and cod liver oil should be added to the diet as a good source of Vitamin A [9]. Drinking water must be available at all times and molasses water or brown sugar water solution could also be utilized. Although they lived near water sources, their main source of water was from the diet and only sought water when chased [12].

Agoutis used the contents of the endocarps (larvae and seed) when the availability of other fruits and seeds were low. Since bruchid larvae was found inside the endocarp (the highest numbers found just after the peak fruiting season), it was concluded that they consumed many bruchid larvae as well [20]. Consumption of animal matter was observed when a female agouti reintroduced into the wild was observed consuming the carrion of a tapiti (*Sylvilagus brasiliensis*)*.* It did so by adopting the regular posture of sitting on its haunches and using its front paws to rip away pieces of meat which it placed into its mouth [21] (Table 1). The agouti eats by assuming a sitting position while using its front paws to hold food [9,12]. Agoutis were observed as short seed dispersers and this act of scatter-hoarding ensured a sufficient supply of food. This makes them essential in the planting of forgotten seed in the forest [15]. The adaptation in diet reflected their scatter-hoarding behavior [13]. They are known to bury food and hoard food so that in times of food scarcity there would be a reliable source [12,18,22].

### 2.2. Structure of the Digestive System

The mouth of the agouti was divided into the vestibule, which contained cheek pouches, and the mouth cavity with a diastema between the incisors and premolars [23]. The dental formula is shown in Table 2. The incisors’ anatomy was rootless and continuously growing, with enamel only on the anterior surface, which allowed the teeth to be chisel-shaped and sharp and it improved the resistance to the stress of biting. The jaw could move forward and create two positions: one where the incisors occluded while the cheek teeth did not and another where the cheek teeth occluded but the incisors did not [24]. The agouti’s (*D. azarae*) premolars had a deciduous form followed by the permanent premolars, whereas molars had no deciduous form [25]. The same dental formula for an adult agouti was found (*D. prymnolopha*) with the teeth of the lower jaw larger than their corresponding upper ones [26].

The agouti (*D. aguti*) had an elongated, triangular shaped tongue containing four types of papillae (filiform, fungiform, foliate and vallate) with varying distributions [27]. On gross anatomy, the tongue was seen as a large, pink, spatula-shaped organ. The esophagus measured, on average, 15.4 cm and had almost a uniform diameter (0.5 cm). The stomach, which had a mean length of 13.8 cm, had a lesser and greater curvature and consisted of the cardia, the fundus, the body and the pylorus [23].

The small intestine of the agouti is long (700.2 cm), which formed 49.1% of the total weight of the entire digestive tract. The duodenum, jejunum and ileum were not clearly demarcated and appeared as a small, coiled mass. The pancreas was found in the sigmoid flexure of the duodenum. The large intestine (which consisted of a large caecum, colon and rectum) formed 36% of the total weight of the digestive tract. The colon and rectum had a mean length of 117.0 cm and formed 18.7% of the total weight of the digestive tract. There were also two large anal glands which opened into the anus. The total digestive tract was 3.15% of the animal’s body weight [23]. Due to their gastrointestinal tract anatomy, the agouti should be able to digest higher levels of protein and soluble carbohydrates than rabbits. The agouti practiced coprophagy, which is the act of ingesting fecal matter, which may contain nutrients not absorbed via the large intestine [9].

## 3. Lappe/Paca (*Cuniculus paca*/*Agouti paca*)

### 3.1. Eating Habits and Feed Type

The paca was described as a strict vegetarian that consumed plants, herbs, nuts, fruits, seeds, tubers and roots, at night. In captivity, they ate corn, sugar cane, bread and melons, with avocadoes, coconuts and mangoes being their favorite foods [10]. They used a sucking action, with their mouths closed, to empty the internal pouch which may contain soft food. They showed a preference for mango, followed by avocado, melon, papaya, banana, orange, pineapple, tomato, apple, cucumber, carrot and chayote, which showed a positive association with total energy content but a negative correlation with water content [28]. Water rations were obtained by consuming roots, tubers, flowers, fruits, seeds and succulent vegetation [10]. Although they browsed, they were mainly frugivorous and had a role to play in seed dispersal [22] (Table 1). Other authors described them as frugivorous animals that consumed a wide variety of fruits, based on seasonal and local availability [29]. Evaluated stomach contents revealed that fruits made up the majority, with 83.9%, followed by pulps (48.1%), seeds (23.4%), exocarps (10.5%), fiber (8%), leaves (7.3%) and to lesser extent insects. Their diet varied during the year, with a high consumption of exocarp and pulp during the main fruiting season; during August–October, there was a high consumption of seeds and leaves/fiber; and exocarp was consumed during August–January, rather than leaves. They consumed more leaves and fiber during periods of least and of increasing fruiting [14].

During gestation, females consumed more exocarp and seeds. They used incisor marks (>0.4 cm) as a criterion to identify food consumed by pacas and concluded that they used thirty-three plant species and consumed fruits (including the skin), pulp, seeds, endosperm, flowers and seedlings. Ants and lepidopteran larvae were also found in fecal samples [30]. They were observed as nocturnal frugivores that fed according to fruit availability and consumed either the entire fruit or rejected the exocarp or seeds [31]. When fruit was scarce, they supplemented their diet by consuming large amounts of leaves. In captivity, they consumed food that humans ate which included fish and other meat (cooked or uncooked) in addition to fruits [32] (Table 1). Dry feed was always placed hours before fruits or seeds and new foods were introduced gradually, in small amounts mixed in with their old feed. Calcium, mineral and vitamin should also be supplemented [32]. Food was usually served in square metal bowls, about 20 cm, and placed in dry areas. In the wild, feed was soiled and therefore in captivity a small amount of soil should be given [32].

In captivity, farmers overfed protein to pacas, as comparable growth rates could be achieved on lower protein diets. The daily requirement of nitrogen was determined to be 280.5 N/kg^0.75^. Thus, finishing pacas required at least 55 g crude protein/kg dry matter and 13 MJ/kg of digestible energy [33]. In the growth phase, 12% protein in the diet was sufficient. The best yield in the average daily weight gain was attained by using a combination of rabbit concentrate (10% protein) and vegetable protein (15% protein) [34]. 

The paca does not use their front paws to aid in feeding [10]. However, some authors noted the paca feeding using its front paws [30]. They deduced that pacas may be important seed dispersers as a high number of seeds was found in fecal samples and the digestive tracts. They carried their food into dens to consume, especially when there was a food dispute. To consume certain food items that they peeled, e.g. ears of green corn (not fruits), they used one or both front paws to hold the item against the floor. In some fruits, they made a small hole with their incisors and consumed the pulp by scraping with the lower incisors [35]. They smelt various food items presented to them before choosing one. These animals intensively sniffed and picked up the preferred food, with its teeth, before returning to their burrows, suggesting that they depended on olfactory information to feed [28].

### 3.2. Structure of the Digestive System

The paca has longer, more sinuous shaped lower incisors as compared to the upper incisors which were smaller and straight. The enamel, in adults, appeared yellow tinged. There was a diastema between the incisors and the premolars. The premolars and molars had a slanted occlusal surface [36]. The dental formula was given (refer to Table 2 [22,36]). The incisors grew continuously throughout life, as there were no roots, therefore in captivity bark or branches should be placed in enclosures to aid in filing them down [32]. The action of gnawing was needed to wear down their incisors [35].

The stomach, which was found closer to the left side in the abdominal cavity, was a unicavity organ with a greater and lesser curvature and consisted of the cardia (pinkish to white in color), the body (brownish in color) and the pylorus (light pink). Microscopic evaluation showed that the mucosa was part aglandular and part glandular [37]. The duodenum was relatively short (average 33 cm) and made up 3.6% of the total intestinal length. The large intestine (cecum, colon and rectum) was located in the abdominal and pelvic cavity. The cecum, found after the ileum, had a saccular shape, was distended at the beginning and ended with a small apex. The colon started around the umbilical region and continued cranially, then curved to the right, following the right lateral region caudally to the pubic area, then took a dorsal position cranially, forming a spiral in the right lateral area. It continued to the left inguinal area, into the pelvic cavity. The ileum was located between the cecum and colon. The rectum was found in the pelvic cavity [38]. Ultrasonography showed that the paca’s stomach exhibited peristaltic movements and it also allowed for visualization of the intestinal silhouettes [39].

The practice of caecotrophy or coprophagy by the paca was unknown [29]. Recently, observations were made where caecotrophy was exhibited. They assumed a sternal position and placed their snouts between the hind-limbs to lick the anus followed by them lifting their heads, chewing and swallowing [35] (Table 1). Two types of feces are produced: one which consisted of hard, dark balls and another which was a soft paste that was ingested during caecotrophy [32].

## 4. Capybara (*Hydrochoerus hydrochaeris*)

### 4.1. Eating Habits and Feed Type

The capybara was observed as being a herbivorous animal which inhabits semi-aquatic environments [11,40]. Authors described them as selective herbivores, as capybaras from the Lower Delta of the Paraná River selected plants according to caloric energy content [41] (Table 1). Their findings supported the optimal foraging theory, rather than the nutritional-benefits hypothesis. In seasonally flooded savannas of Venezuela, they were observed to select more profitable food items (with respect to protein and fiber content) when a greater variety was present during the wet season, which was also in accordance with the optimal foraging theory [42]. Capybaras have been described as both grazers and browsers that showed an increase in dry weight of dicotyledons during the dry season [43].

With changing availability of food resources, they adjusted their foraging patterns [11]. In the agricultural landscape in Brazil, they consumed rice the most, followed by sugarcane, with soybean being the least consumed. It was assumed that, due to human activities, the capybaras fed during the evening to night periods and preferred grazing on areas where they could make quick escapes, thus they avoided the open pastures [44]. Capybaras were also found in open areas with sugar cane and cultivated pasture [45]. Open wetland area in southeastern Brazil, with profuse forage, had the most number of capybaras, as compared to the forested area and mixed agricultural fields [46].

Habitats suitable for capybaras, in Argentina, consisted of areas with grasses or sedges less than 100 cm (e.g., *malezales*, grasslands and prairies) which had the highest forage values [47]. Plant species collection and fecal analysis showed only 14.8% of terrestrial plants were found in the diet, with aquatic plants making up the majority (87%) [48]. Capybaras were more prevalent where water was readily available [44,45]. Aquatic feeding took place during the day; capybaras swam to the feeding area (where they stayed for 30–80 min), dove underwater to collect the seagrass and re-surfaced to chew it. *Ruppia maritima* was the only submerged vegetation in the study area and the capybaras did not show preference for any specific part of the seagrass [49].

The capybara has been described as a large (30–50 kg) herbivorous hindgut fermenter, with active microbial fermentation in the caecum [50]. When compared to other livestock, their digestibility of the same feedstuff was higher, which could be due to their anatomical and physiological characteristics [51]. Lower levels of nitrogen, calcium and phosphorus caused decreased weight gains and the levels of nitrogen, calcium and phosphorus required for maintenance of the capybara was lower than those required by other species [52]. Capybaras suffered from scurvy, as, during vitamin C deprivation they displayed signs of scurvy, such as broken or loss of incisors, gingivitis and even one death, which started 25–104 days after deprivation. These signs also appeared when the diet consisted mostly of pelleted rations and slower growth rates were observed [53].

### 4.2. Structure of the Digestive System

The dental formula of the capybara is shown in Table 2 [22]. Their cheek teeth are complex teeth that grow throughout life and are multilaminated [40,54]. The capybara has a simple stomach, which consisted of a cardia, pylorus, body, fundus and gastric diverticulum [55]. The small intestine consisted of the duodenum (ascending and descending), jejunum and ileum and was approximately twelve times the animal’s body length. The female’s small intestine ranged 441.0–1734.0 cm and the male’s ranged 355.0–1123.0 cm. The border between the duodenum and jejunum was demarcated, while the border between the jejunum and ileum was not. The duodenum made up 2.5% of the total length of the small intestine, while the ileum made up 2.4% [56]. The large intestine was divided into the caecum (largest part; divided into a base, body and apex), colon (ascending, transverse and descending), rectum and anal canal [57].

The capybara is the largest mammalian herbivore to practice coprophagy [58] and/or caecotrophy [52]. Caecotrophy was observed, more so in the mornings than at nights; animals sat on their hindlimbs and extended either one of them. They would then bend their heads down, placing their snouts by their anus, to lick the pasty feces and then return to an upright position to chew and swallow [59,60]. In capybaras fed a diet of solely grass, caecotrophy was observed more often and they showed the lowest daily weight gains. By introducing grain corn into the diet, the daily weight gains were the highest, the feed conversion improved and caecotrophy decreased [60]. In addition to protein, energy, mineral and vitamin supplementation, good quality roughage should also be provided to stimulate caecotrophy [61]. 

## 5. Manicou (*Didelphis marsupialis inularis*)

### 5.1. Eating Habits and Feed Type

*Didelphis* spp. has been classified as adaptable omnivores, with a predilection for animal matter [62]. *D. virginiana* and *D. marsupialis* diets included insects, small vertebrates, fruits and leaves, while *D. albiventris* consumed fruits, small vertebrates, bird eggs and invertebrates [63]. *D. marsupialis* consumed flowers, while animal prey varied according to species. Insects predominated and other food consumed included worms, frogs and carrion [64,65]. *D. aurita* showed no food preference and ate a variety of plants and invertebrates, such as beetles, ants and butterflies [66].

Seasonal variations in the diet were seen in the *D. marsupialis* [67], in the *D. virginiana* in Virginia [68] and in the *D. virginiana* in Portland [69]. The feeding habits of 108 opossums (*D. marsupialis*) were observed for one year in Venezuela and it was found that mammals, birds, insects and gastropods were consumed during the dry season, as compared to the wet season, where fruits were of greater importance, followed by birds, mammals, insects, snakes, toads and earthworms [67]. Throughout the year, however, food from animal sources comprised more (63.5%) of the diets than plant species (22.9%). The proportionate annual volumes of 102 stomachs showed that birds made up the most with 21.5%, followed by mammals (15.3%), insects (14.8%), fruits (12.8%), particulate material (11.6%), plant remains (9.4%), carrion (5.0%), gastropods (2.5%), garbage (2.0%), toads (1.6%), centipedes (1.5%), earthworms (1.0%), grass (0.7%) and snakes (0.3%). In terms of frequency, insects contributed the most to the annual diet with 49.1%, followed by fruits (18.6%), birds (12.7%) and mammals (8.8%). Younger animals consumed mainly invertebrates, fruits and plant material, while the adults consumed these in addition to mammals and birds [67].

The stomach contents of 77 opossums (*D. virginiana*) in Portland (December–May) were evaluated, finding that mammals predominated during winter and spring; beetles, slugs and snails during the summer; and fruits during the summer and fall. Earthworms did not show any seasonal trends [69]. The stomach contents of 129 *D. virginiana virginiana* (samples were taken from the warmer months (May–October)) were evaluated, finding that insects made up the majority with 59.7% occurrence followed by fruits (49.7%), amphibia (36.4%), mammals (32.6%), grasses (30.2%), worms (20.3%), mollusks (18.6%), reptiles (19.4%), birds (13.2%), carrion (7.0%), grain (2.3%), undetermined plants (6.2%), undetermined animals (6.2%), centipedes (1.6%), millipedes (0.8%) and fungi (0.8%) [70,71]. In captivity, the priority was to maintain the proper calcium:phosphorus ratio and to avoid high fat meals. In addition to their natural diets, cat and dog food could be added, along with a fresh supply of water. Healthy adults did not need vitamin supplementation. Juveniles could be fed a mash and gradually introduced to soft food, then small pieces of meat and fresh water as they got older or gained weight [68].

*D. albiventris* was observed as a seed disperser. Its diet consisted of mainly invertebrates (Arthropoda, 34.9%; and Molusca, 7.8%) and fruits (28.8%) followed by vertebrates (bird, 10.8%; mammal, 10.5%; and fish, 7.2%) [54,64]. The diets of 12 species of Didelphidae were investigated and species with more carnivorous diets had high protein contents, while the more frugivorous species showed high non-structural carbohydrates quantities [72]. *D. marsupialis virginiana* was given various food items and it was noticed that, although they consumed all, they showed a preference for animal matter, including the poisonous *Bufowood houseifowleri* and *B. americanus* [73]. Olfaction was the most important sense to them when they hunted non-living food items, while vision and hearing played a bigger role with live prey. Since smaller prey items easily escaped them, it was concluded that they were not very efficient predators of mammals in the wild [74]. *D. marsupialis* was observed to predate on rattlesnake (*Crotalus durissus*), where they would either immobilize them with quick, consecutive bites along the body, followed by consumption (starting from either end) or consume them alive (starting from the tail) if they were immobilized, or chew the head when attacked [75]. To eat, they sat and alternated holding the snake with their forelimbs, occasionally using both to tear pieces. The snake was placed into the mouth laterally and chewed. No effects of being envenomated were observed [75]. *D. marsupialis*, with five young in her marsupium, attacked, killed and consumed another opossum, *Philander opossum* [76]. Newly weaned litters, in the same cage, showed cannibalism [76]. They drank 200.0–500.0 mL of water/day and that consumption was two to three times more during the summer than winter [74].

### 5.2. Structure of the Digestive System

There is some discord between authors with respect to the dental formula (refer to Table 2). The anatomy of the digestive system of *D. marsupialis insularis* was similar to that of most other omnivorous marsupials, with a well-developed caecum [62]. *D. marsupialis* has a wide mouth with a shorter lower jaw and small, pointed canines [77]. The opossum’s (*D. marsupialis*) tongue was divided into a posterior fixed root, an anterior free tip and a fixed lingual body (between the root and tip). It averaged 5.9 in length, 3.3 cm in width at the lingual body and 3.8 cm at the root [78]. The ventral tongue surface was smooth, while the dorsal surface was not due to papillae—filiform (sharp filiform distributed across the entire tongue; conical filiform on the lingual body and tip), fungiform (scattered among the filiform papillae on the lingual body and tip) and vallate (three at the root) [78]. Strands of papillary projections on the root, facing the oropharynx, was a unique feature of the tongue of the *D. marsupialis*, as compared to the other opossum species [78]. The stomach is simple, globular and the caecum is almost 20–40% of the total body length, and is simple and conical [68].

Digestive tracts of seven Didelphid marsupials was examined and it was found that they differed significantly among species; the caecum varied the most, with an average coefficient of variation of 36%, followed by the hard gut (23%), esophagus length (20%), stomach (16%) and then the small gut, which was the longest segment, varied the least (8%) [79]. They found that *D. albiventris* esophagus measured 15.8 cm in length, the stomach was 9.1 cm, the small intestine was 114.9 cm, the caecum was 7.8 cm and the hard gut was 30.4 cm [79]. They also examined another species, *D. aurita,* and found that the esophagus measured 14.2 cm in length, the stomach was 7.6 cm, the small intestine was 117.7 cm, the caecum was 7.5 cm and the hard gut 33.6 cm [79].

## 6. Collared Peccary (*Pecari tajacu*)

### 6.1. Eating Habits and Feed Type

Collared peccaries were described as slow, graceful eaters which utilized their feet to hold down food items. They held the prickly pear pads against the ground with the front feet and peeled the skin off from one side, to reach the pulp [80].

They are described as being frugivorous and seed predators, which disperse small seeds. Their diet consisted mainly of fruits, with several seeds found in the stomach, along with very small amounts of insect pupae and larvae [81,82]. They eat fruits and gathered in groups when locally abundant food was present [83]. Fruits comprised 78% of their diets in the dry and wet seasons. Although fruit diversity was lower in the dry season as compared to the wet season, they consumed more in the dry season (2.94 g/m^2^/month) than wet season (1.40 g/m^2^/month). There was also higher tuber consumption in the dry season (63%) as compared to the wet season (35%). Some seeds consumed were still intact, thus could be dispersed [84]. They could be found in a wide range of habitats, thus making their diets very diverse. Some primary food items, according to location, included fruits, underground tubers, rhizomes, bulbs, acorns, green grass, green shoots of annual plants, fruits and cladophylls (stems) of prickly pear cactus, and succulent agaves (Table 1) [80].

The annual diet of peccaries in south Texas consisted of cacti (74.7%), woody plants (15.3%), forbs (5.1%), grasses (2.3%), unknown plants (2.3%) and animal matter (0.3%), with pricklypear cactus (*Opuntia lindheimeri*) and honey mesquite pods (*Prosopis glandulosa*) being the two most important plants, making up 86.3% of the diet [85]. Availability affected their diets; the pricklypear pads made up the majority of the diet October–March, during the fall and winter; during the spring and summer pricklypear fruit made up the majority of the diet from April until the summer when mature mesquite pods were available and then the diet consisted of both of these food items (until September) and by October, when their numbers decreased, the peccaries switched back to the pricklypear pads [85]. Annually, forb (*Portulaca mundula*) and pricklypear made up 69.1% of their diet during the fall and early winter [85]. The diet of the collared peccary varied seasonally. In May, July and August, pricklypear cactus dominated their diet. During October and March, forbs, in addition to pricklypear cactus, were equally present. In January, forbs dominated the diet [86,87].

Protein was important in their diet as animals lost weight when no protein was fed, but they maintained their weight on a 5% protein mix, with weight gains on 10% and 15% protein levels [80]. Experiments also showed that Vitamin B complex was a deficiency in a cactus diet as peccaries became weak and emaciated after prolonged use of such a diet, which was corrected by intramuscular injections of Vitamin B complex [80]. However, based on the physical conditions of the two-month-old experimental animals, no Vitamin B deficiency occurred and there was no difference in weight gains of those fed rations supplemented with Vitamin B and those not supplemented. It was postulated that coprophagy supplied the vitamin B requirement [88].

They were comparable to other herbivores with respect to digesting fiber [89]. Dietary fiber decreased the digestibility and they responded by increasing dry matter intake to maintain their energy balance. Their digestive efficiency of fiber was equal to that of a ruminant, the deer, and this was believed to be due to the long mean digesta retention time. This slower rate of digesta passage due to the unique anatomy of their stomach increased the digestive efficiency [90]. Their ability to digest fiber fell between monogastrics and true ruminants [88]. They digested fiber better than domestic swine, but not as efficient as true ruminants, with an average fiber digestion of 36.5% [88]. Their ability to digest fiber was closer to that of the domestic swine than domestic or wild ruminants [91]. Based on an all alfalfa hay ration, they could tolerate a high cellulose diet for a limited time [88].

Compared to a similar sized hindgut fermenter, on a similar diet, their digestibility of dry matter, energy and fiber did not provide a significant benefit [91]. Collared peccaries, as compared to small hindgut fermenters, had a higher digestibility of fiber [92]. They had a lower fermentative ability and forage digestibility due to their relatively small stomach volume when compared to other foregut fermenters [93].

In one experiment, the dry matter intake (g/kg) for treatment group A (citrus meal, green banana fruit and dasheen tubers), treatment group B (*Tricanthera gigantea* (leaves and stems), coconut meal and pig grower) and treatment group C (soybean meal, pumpkin and cassava tubers) were 23.2, 24.3 and 31.2, respectively [94]. Preference index analysis of each food item showed that green banana, cassava tubers, *Tricanthera gigantea* (leaves and stems) and pumpkin were the most preferred, while soybean meal, coconut meal and citrus meal were the least preferred. It was concluded that there was no correlation between crude protein intake and dry matter intake [94].

Dry matter digestibility was higher for concentrate (84%) than for natural diets (49–72%) and they consumed more concentrate during the cooler months [95]. Diet digestibility was directly related to nitrogen and animal’s age but inversely related to consumption, gross energy, phosphorus, fiber and ash [95]. Non-protein nitrogen experiment was not conclusive as results (intake) were low in both treatment groups (one treatment group used urea as the nitrogen source while the other used soybean meal) and weight loss was constant [96].

During the summer, water requirements for collared peccaries were 66.5 mL/kg/day and, during the winter, 38.6 mL/kg/day [97]. Their total daily energy requirement was found to be 794.1 Kcal/day in the summer and 917 Kcal/day in the winter, for an 18.2 kg collared peccary, with an annual energy requirement of 17,166 Kcal [97]. The collared peccary in Paraguay consumed the roots of *Boerhavia coccinea* and consumption was greatest in fall and winter [80]. They were observed as diurnal animals and found that weight gain was better on a diet of 40% babassu meal (*Orbignyapha lerata*) than corn, which provided a cheaper alternative than corn [98]. *Guazu maulmifolia* forage which had high moisture content (34.98%) was the most consumed followed by *Leucaena leucocephala* and *Brosimuma licastrum,* with *Pennisetum purpureum* being consumed the least [95]. These three latter forages had high amounts of crude protein and tannins, thus could be used for captive collared peccaries in addition to fruits, to supplement protein [95]. On a commercial swine diet, as compared to a purified diet, collared peccaries had a higher feed intake and consumed more water [94].

The average daily consumption of green cactus was 3.9 kg maximum. The water content of cactus collected from Tucson averaged 82.6%, thus the total daily water intake during the summer could reach 3.0 L [80]. Therefore, they could meet their water requirements on a diet of mostly green cactus [80,96]. Captive collared peccaries had a turnover rate of 1.58 L of water a day, similar to free-living ones, even though their total body water was higher [99].

### 6.2. Structure of the Digestive System

The dental formula at birth and for adults is shown in Table 2. At birth, all four temporary canine teeth and two lower incisors were present [17]. Their large, sharp canine teeth (which grow until they are about four years old) are used as a defense mechanism rather than having a role to play in their diet, as they were not carnivores [80]. They used their snouts to dig for bulbs, roots and tubers [80]. They had long, interlocking canine teeth and could not move their jaws sideways. The incisors were adapted to consuming vegetation. The constant contact of both upper and lower canines ensured that they were always sharp [22]. 

The unique feature of the peccary’s stomach is that it consisted of a fundus that had an extension resembling an abomasum, and two blind pouches on either side [80,88]. It was postulated that they could digest forage based on the size of the caecum and large intestine [88]. The stomach was present transversely within the abdominal area. It consisted of three gastric pouches, with indistinct boundaries, and a glandular stomach which had a greater and lesser curvature [90].

Peccary stomachs were analyzed and based on the pH of the digestive tract it was possible for them to synthesize volatile fatty acids from cellulose [80]. The pH of what was referred to as the rumen (which was the fundic portion with the two wings) was suitable for microbial population (6.2) [88]. Their digestive tract had a low concentration of volatile fatty acid, with the highest concentration being found in the rumen and lowest concentration found in the caecum and anterior large intestine [88]. The total volatile fatty acid concentration was 55 µm/mL, which was similar to the rumen [88,100,101].

## 7. Red Brocket Deer (*Mazama americana*)

### 7.1. Eating Habits and Feed Type

Red brocket deer have been described as browsers and frugivores [22], while others have classified them as ruminants that consumed fruits, fungi, browse (mainly when fruits were scarce) and fallen flowers [65]. *Mazama americana trinitatis* diet consisted of grasses, vines and tender green shoots [102] (Table 1). They consumed various seeds, including the hard palm seeds of *Iriartea* spp., *Euterpe* spp. and *Mauritia flexuosa*, due to them being ruminants; with the first two seeds present in 59% of samples [103]. They consumed fruits more than leaf or fiber, with rumen samples consisting of 81% fruit [103,104]. They showed adaptation in their diet, as during flooded periods in the Amazon basin samples contained greater amounts of leaf or fiber and fewer fruits [105,106]. Thus, red brocket deer are classified as being frugivorous ruminants and that rumination allowed them to consume seeds after the initial break-down by rumen microbes [105].

Red brocket deer was described as a frugivore-granivore that showed little variation in their diet throughout the year (Table 1). They consumed fruits and seeds the most (56%), followed by fibers (24%), leaves (13%), flowers (5%), fungi (0.6%) and animal matter (0.5%) [107]. Authors have disagreed stating that they were herbivores, with no animal remains found in any samples [108]. During the fruiting season (February–May), fruits and seeds were consumed the most, while, during fruit scarcity, they consumed more leaves and fiber (June–September, with fiber being consumed throughout the year), while the majority of flowers and fungi were consumed during October–January and animal matter showed no seasonal inclination [107]. In Suriname, the diet changed seasonally with rainfall. The57 rumens that were examined contained 57 plant species, with shelf fungi making up 45% of the diet in July. Different fungi species combined were the most abundant food item for any given month. Fungi were consumed the most during the long-wet season and the least during October (driest month) [109]. The aggregate percent volume of seeds, fruits and flowers was 56% with a high consumption of fruits and seeds in December and the relationship between consumption and rainfall was unclear. Analysis of rumen bypass of fiber was done with them digesting more hemicellulose than cellulose. *M. americana* was found to consume, as a percentage of dry matter, 16.2% crude protein, 34.0% neutral-detergent fiber, 17.5% hemicellulose, 13.0% cellulose and 3.3% sulfuric acid lignin [110]. *Mazama* spp. had the slowest gastro-intestinal transit time, which gave them a better ability to digest fiber [111]. *Mazama* spp. frequented mineral licks, but during the day at the hunted site were less active as compared to the non-hunted site [112,113]. Dry matter digestibility increased by supplementing with mineral blocks [114].

### 7.2. Structure of the Digestive System

The dental formula for Cervidae and *M. americana trinitatis* is shown in Table 2. Canines were present in young animals, being part of their milk dentition, and not in mature animals, except for three adult animals [115]. The tongue was covered by different types of papillae. The apex, body and lingual torus of the tongue contained a mix of filiform and fungiform papillae. The root of the tongue had a double row of vallate papillae on the sides [116].

The gross anatomy of the gastrointestinal tract of the brown brocket deer (*M. gouazoubira*) was evaluated and it was found that the stomach had four chambers, consistent with other ruminants [117]. The pH of the rumen was found to be 6–8 and the pH of the abomasum was 4.9 [103]. The ascending colon had a proximal S-shaped ansa, a spiral ansa (short; included 1.5 centripetal gyri, a central flexure and 1.5 centrifugal gyri) and a distal ansa. The small intestine:gross intestine ratio was 2.0 which, in conjunction with the other gross findings, placed it within the browser range [117].

## 8. Conclusions

In conclusion, neo-tropical animals that are present in Trinidad have various diets. These animals are adapted to our environment and thus can be fed intensively using local feedstuff. Some are caecotrophic and hindgut digesters such as the agouti, lappe and capybara, allowing them to utilize fiber and convert it into amino acid and vitamins. The manicou is quite unique; it is omnivorous and an opportunistic feeder. The collared peccary has been described as a pseudoruminant and can digest fibrous plant material in its forestomach. This is similar to how fiber is digested in ruminants. The red brocket deer, which is a ruminant, can digest fiber in its rumen similar to domestic cattle.

More work has to be done to evaluate the nutrient requirements of these neo-tropical mammals. Nutritional requirements for each physiological state must be known and formulated rations must be developed. The proper understanding of nutrition of these animals is important if these animals are to be domesticated and reared intensively.

## 9. Future Directions

Even though it appears that there is a lot of information regarding these neo-tropical species, evaluation of the available literature shows that we have barely scratched the surface, as compared to information known for domesticated species. The nutrient requirements for these animals at different physiological stages need to be investigated and nutrient requirement tables established. Therefore, one of our recommendations will be to conduct feed trials. However, even before reaching that stage of nutrition, we need to increase the sample sizes of these named neo-tropical species to evaluate their digestive systems thoroughly. When we fully understand how they feed in the wild (inclusive of the dentition and digestive system), we will be better able to care for them in captivity. When this is established, the next step will be to create well-balanced feed rations and supplemental feeding formulae for the young, orphaned animals.

## Figures and Tables

**Table 1:** Diet and feed type of common neo-tropical mammals.

Animal	Diet	Type	Reference
Agouti	Roots, tubers, ferns, succulent plants, bark, fruits, berries, seed and nuts (Caecotrophy), animal matter	Herbivore, Vegetation, Opportunistic feeders	[10,12,21,28]
Lappe	Mango, avocado, melon, papaya, banana, orange, pineapple (Caecotrophy)	Opportunistic feeder	[28]
		Frugivore	[22,28]
Capybara	Plants (Caecotrophy)	Herbivore (hindgut fermenter)	[11,41,50,58]
Manicou	Insects, small vertebrates, fruits, seeds, leaves, eggs	Omnivore	[62,63,64,65]
Collared peccary	Fruits, tubers, rhizomes, plants, prickly pear cactus	Frugivore (pseudoruminant)	[80,82,83,101]
Red Brocket Deer	Fruits, fungi, browse, flowers, grasses, vines and tender shoots	Browsers and Ruminant Frugivores	[22,65,102,103,107]

**Table 2:** Dental formula of common Neo-Tropical Mammals.

Name	Dental Formula	Reference
Agouti	I 1/1, C 0/0, P 1/1, M 3/3 = 20	[22,23]
Lappe	I 1/1, C 0/0, P 1/1, M 3/3 = 20	[22]
	I 1/1, C 0/1, P 1/1, M 3/3 = 22	[32]
Capybara	I 1/1, C 0/0, P 1/1, M 3/3 = 20	[22]
Manicou	I 5/4, C 1/1, P 3/3, M 4/4 = 50	[68]
	I 5/4, C 1/1, P 2/3, M 4/4 = 48	[22]
Collared peccary	I 0/1, C 1/1, P 0/0, M 0/0 = 6 (Birth)	[12,84,101]
	I 2/3, C 1/1, P 3/3, M 3/3 = 38 (Adult)	
Red brocket deer	I 0/3, C 0-1/0-1, P 3/3, M 3/3 = 32 or 34	[65]
	I 0/3, C 0-1/1, P 3/3, M 3/3 = 32 or 34	[102]
